# Daily routines, short-term priorities, and nurses’ role hamper self-management support in a hospital setting: A mixed methods study

**DOI:** 10.1016/j.ijnsa.2024.100279

**Published:** 2024-12-05

**Authors:** Susanne van Hooft, Elke Berger, Cailey van Torenburg, AnneLoes van Staa

**Affiliations:** a Research Centre Innovations in Care, Rotterdam University of Applied Sciences, P.O Box 25035, 3001 HA Rotterdam, the Netherlands; b Franciscus Academy, Franciscus Gasthuis & Vlietland, Kleiweg 500, 3045 PM, Rotterdam, the Netherlands; c Pulmonary and Cardiology ward, Franciscus Gasthuis & Vlietland, Vlietlandplein 2, 3118 JH Schiedam, the Netherlands

**Keywords:** Chronic disease, Hospitals, Nursing, Self-management, Questionnaires

## Abstract

**Background:**

Self-management support is widely considered a critical aspect of nursing. Still, many studies indicate that nurses frequently experience difficulties in daily practice.

**Objective:**

To gain a deeper understanding of the factors perceived by nurses to impede or promote their support of patients’ self-management within the dynamic environment of the in-patient hospital setting.

**Design:**

Mixed methods design.

**Participants:**

Nurses (*n* = 269) working in a teaching hospital in the Netherlands completed a questionnaire. Subsequently, 38 nurses participated in interviews.

**Methods:**

The SEPSS-36 questionnaire assessed nurses’ self-efficacy and performance in self-management support. Semi-structured interviews were conducted to address salient results from the questionnaire, focusing on factors influencing self-management support, goal setting, follow-up care, and the nurse's role in a hospital setting.

**Results:**

the response rate for the questionnaire study was 62 %. A paired t-test revealed a significant mean difference of 6.30 95 % CI [5.91–6.69] *p* ≤ 0.001 between nurses’ perceived self-efficacy (mean = 18.34/24) and their actual performance (mean = 12.03/24) in self-management support. The interviews revealed that nurses typically focus on medical procedures and maintaining patients’ functional status. Spending time with patients to offer emotional support was viewed as ‘something extra’ rather than a core part of their job. High patient turnover hindered nurses from having meaningful conversations with patients.

**Conclusions:**

Short-term priorities such as ‘getting the work done’ dominate nurses’ daily tasks in hospital wards, leading them to overlook often the benefits of supporting patient self-management. This narrow view of their responsibilities can hinder patient care, whereas adopting a broader perspective on the patient journey could be very beneficial.

**What is already known**

Adequate self-management offers benefits such as improved quality of life, greater independence from healthcare professionals, more empowerment, better shared decision-making, and reduced hospital admissions.

Self-management support is an aspect of nursing. But, many nurses struggle to fully support patients’ self-management.

Despite recognising holistic care as essential for high-quality care, it is unclear why nurses do not provide this during hospital stay.

Alt-text: Unlabelled box

**What this paper adds**

Nurses tend to work with a mental checklist, prioritising functional tasks such as ambulation, eating, hygiene, and wound care. Spending supportive time with patients is often considered a non-essential task.

The provision of self-management support is also influenced by differing mutual expectations of patients and nurses regarding each other's roles. These are not discussed during patient admission.

A perceived lack of time is not the only constraint in supporting self-management. The nurses’ mindset is focused on short-term priorities, making it more challenging to address emotional or role management issues.

Alt-text: Unlabelled box

## 1 Background

Self-management is pivotal for the management of long-term conditions. Self-management is defined as “the individual's ability to manage symptoms, treatment, physical and psychosocial consequences and lifestyle changes inherent in living with a chronic condition and to affect the cognitive, behavioural and emotional responses necessary to maintain a satisfactory quality of life” (Barlow et al., 2002, p. 178). This involves focusing alternately or simultaneously on three domains of self-management: medical management, role management, and emotional management (Lorig and Holman, 2003). Medical management entails handling the therapeutic regimen, role management involves integrating the condition into daily activities, and emotional management requires coping with the uncertainty of living with the condition (Lorig and Holman, 2003). Adequate self-management offers benefits such as improved quality of life, greater independence from healthcare professionals, more empowerment, better shared decision-making, and reduced hospital admissions (Kendall et al., 2011).

When individuals lack the capability or knowledge to manage their health independently, they may need support from healthcare professionals. The Five A's model clarifies the process of self-management support. This model distinguishes five phases: Assess (assessing the patient's motivation and beliefs), Advise (providing information and instruction), Agree (setting mutual goals), Assist (helping the patient overcome barriers), and Arrange (follow-up care and coordination) (Glasgow et al., 2006; Whitlock et al., 2002).

Nurses often play a crucial role in providing this self-management support due to their approachable contact with patients (Elissen et al., 2013). Duprez et al.'s construction of a list of competencies clarified nurses' roles in self-management support. This list of competencies was structured according to the previously mentioned Five A's model, but an additional sixth category for overall competencies in self-management support was included (Duprez et al., 2016). However, many nurses struggle to fully support self-management across all three domains (van Hooft et al., 2016). Research indicates that nurses tend to focus more on medical management and daily routines, often paying less attention to role management and emotional management aspects of self-management (Been‐Dahmen et al., 2015; van Hooft et al., 2018). Additionally, nurses primarily aim to help patients become self-reliant in their daily activities (Otter et al., 2021). Despite recognising holistic care as essential for high-quality care (Stavropoulou et al., 2022), it is unclear why nurses prioritise functional tasks, such as ambulation, eating, hygiene, and wound care.

When seeking explanations for nurses’ self-management support behaviour, self-efficacy, defined as an individual's belief in their capacity to perform certain behaviour, should be considered (Bandura, 2006; Kosmala-Anderson et al., 2010). Research shows that nurses are often uncertain about what self-management support entails (Otter et al., 2021). Other possible explanations influencing performance in self-management support relate to hospital-specific factors such as high patient turnover, shift work, and hierarchical structures (Brand et al., 2012; Rosnawati Muhamad et al., 2021). Recent systematic reviews have identified several factors that influence the implementation of self-management support, including time constraints, nurses' knowledge and skills, and patient-related factors, such as motivation and patient needs (Noordman et al., 2023; Tharani et al., 2021). Still, these do not satisfactorily explain the trade-offs nurses make in daily practice about supporting self-management holistically. In this study, our objective is to gain a deeper understanding of the factors perceived by nurses to impede or promote their support of patients’ self-management within the dynamic environment of the in-patient hospital setting.

## 2 Methods

### 2.1 Design

A sequential, explorative, cross-sectional, mixed-method design was used to identify nurses' self-management support practices during inpatient hospital care. The study comprised two parts. First, a questionnaire was administered to evaluate nurses’ self-efficacy and performance in self-management support. Second, individual face-to-face interviews were conducted to explore factors influencing nurses' support of patients’ self-management.

### 2.2 Setting and sample

Data were collected in a teaching hospital in the Netherlands, specifically in wards where nursing students worked and collected data for their bachelor's theses. In total 15 of the 29 hospital wards participated in the study. We employed a total sample approach regarding the questionnaire for the participating wards, inviting all nurses and nurse students working in these 15 wards, either face-to-face or by email. Nurses were eligible for the study if their job description included providing patient care at an inpatient ward. Additionally, a convenience sample of nurses from nine of the 15 participating wards was invited to participate in further interviews. In order to include a diversity of opinions, we encouraged the students to include participants with a broad range in age, education and gender.

### 2.3 Data collection

#### 2.3.1 Procedure

Nurse students (*n* = 15) collected data between February 2022 and May 2023. Before collecting data, the students were given detailed instructions on administering the questionnaires. The nurse students collected the data using a paper version of the questionnaire. Subsequently, they entered the data into a secure system under the supervision of a researcher (EB).

The students received thorough training in interview methods. After the students had conducted their first interview, their transcriptions were provided with feedback from the first author and second authors to refine subsequent interviews.

The local hospital's medical ethics committee stated that this study (2021-044-z) was not subject to the Dutch Medical Research Involving Human Subjects Act. All participants received written information about the study and signed informed consent. Anonymity and confidentially were ensured.

#### 2.3.2 Questionnaire

The questionnaire comprised a validated instrument and demographic data. Specifically, the Self-Efficacy and Performance in Self-management Support (SEPSS-36) scale was used to assess nurses’ self-efficacy and performance in self-management support (Duprez et al., 2016). The SEPSS-36 consists of the essential nursing competencies for supporting self-management, ordered according to the 5A's model: Asses, Advise, Agree, Assist, and Arrange (Whitlock et al., 2002). The Five A's model describes the process of self-management support in five phases. The *Assess* phase involves assessment of the patients’ beliefs and motivations. In the *Advise* phase instruction and information are provided. *Agree*, the third phase, involves mutual goal setting, whereas the fourth phase *Assist* includes overcoming barriers in daily living. The last phase, *Arrange*, involves follow-up care. Duprez et al. (2016) added a sixth category comprising *Overall competencies*, such as a partnership approach or deviating from protocols when deemed necessary.

The 36 competencies of the SEPSS-36 are all assessed with two statements. The first statement *‘I think I can do this’* gauges the extent to which participants believe they can perform this competency (self-efficacy), ranging from “Not at all” (0) to “Good” (4). The second statement ‘*I do this*’ evaluates the extent to which one performs the competency in nursing practice (performance), ranging from “Never” (0) to “Always” (4). The Cronbach's alpha for the measurement of self-efficacy was 0.96; the Cronbach's alpha for the measurement of behaviour was 0.95 in the validation study (Duprez et al. 2016).

#### 2.3.3 Interviews

The qualitative aspect of the study involved conducting in-depth semi-structured interviews using an interview topic guide comprising open-ended questions. An interview protocol was developed by the first author based on the questionnaire outcomes. Three overarching themes guided the interview process: 1) perspectives on providing self-management support (e.g. “*What do you consider to be important in your nursing care?*”), 2) factors influencing self-management support (e.g. “*What is the first thing that pops up in your mind as a cause for not supporting patients’ self-management?*”), and 3) elaboration of the questionnaire survey outcomes (e.g. “*The results of the questionnaire show that nurses do not often set goals together with patients. Do you recognise this in your practice? How could this be improved according to you?*”).

The interviews were audio-recorded and transcribed verbatim.

### 2.4 Data-analysis

#### 2.4.1 Integration of the data

The quantitative data and qualitative data findings were analysed separately, and then integrated by using the qualitative data to explore the quantitative outcomes in more detail (Creswell and Clark, 2011).

#### 2.4.2 Questionnaire

The authors conducted the analysis and generated descriptive data for the demographic characteristics. Means with standard deviation were calculated per subscale and mean sum scores were calculated for the overall scores on self-efficacy and performance. For each of these overall scores, the score range is 0–24, with lower scores implying participants do not have confidence in their self-management support ability (self-efficacy) or that participants do not support self-management in daily practice (performance) and higher scores that they do have the confidence and that they support patients’ self-management on a regular basis.

Differences in the subscale and overall scores between self-efficacy and performance were tested with paired t-tests. Statistical significance was defined as a *p-*value < 0.05. IBM SPSS version 28 was used for statistical analysis.

#### 2.4.3 Interviews

The qualitative analysis was an iterative process (Creswell & Creswell, 2017). The audio recordings of each interview were transcribed verbatim, read through and subsequently inductively coded and categorised into themes first independently by the first and second authors (SH, EB). All authors jointly discussed the themes and interpretation of the data, went back to the data for further analysis to reach a consensus about the most prominent themes: ‘goal setting and aftercare’, ‘routines and priorities’, and ‘the role of nurses in hospital care’. To provide further insight into the questionnaire outcomes, insights retrieved within these three themes were aligned with the six subscales of the questionnaire in a matrix format. This made further categorising and interpretation of data possible.

## 3 Results

### 3.1 Participants

Of the 432 registered nurses invited to participate in the questionnaire study, 269 (62 %) completed the questionnaire. Although nurse students were also invited, they did not participate. Additionally, 38 nurses participated in the interviews. We refer to Table 1 for the characteristics of the participants.

**Table 1:** Characteristics of participants.

**Characteristics**	**Questionnaire n (%)**	**Interviews n (%)**
*Total sample*	269	38
*Age (years)*		
<20	19 (7)	1 (3)
20–30	121 (45)	18 (47)
31–40	51 (19)	6 (15)
41–50	30 (11)	3 (8)
51–60	39 (15)	7 (18)
>61	6 (2)	0
missing	3 (1)	3 (8)
*Work experience (years)*		
<2 years	86 (32)	9 (24)
2 – 5 years	80 (30)	14 (37)
6 – 10 years	24 (9)	6 (15)
11 – 20 years	42 (16)	6 (15)
>21 years	34 (13)	3 (8)
*Educational degree*		
Nurse assistant	4 (1)	1 (3)
Vocational degree	161 (60)	14 (37)
Bachelor degree	98 (36)	19 (50)
Else	3 (1)	
Missing	3 (1)	

### 3.2 Questionnaire: self-efficacy versus performance

The total mean (SD) sum score of self-efficacy was 18.34 (2.66) on a scale of a maximum of 24, implying that nurses were self-confident about their self-management support competencies. The total mean (SD) sum score of performance was 12.03 (3.2), indicating that nurses, on average, performed self-management support activities ‘*sometimes*’ (Table 2). The paired t-test showed a significant difference between self-efficacy and performance for all the subscales (*p* < 0.001) (Table 2). Although nurses seem confident in assessing patients’ preferences, reflected in a mean (SD) of 3.21 (0.51) on the subscale ‘Assess’, the gap with the mean of 2.04 (0.7) on performance for this subscale is the largest of all subscales.

**Table 2:** Overall scores on self-efficacy and performance.

	Self-efficacy		Performance		Mean difference	*p*-value
Subscales (*n*)	Mean	SD	Mean	SD	95 % CI	<0.001
Assess*(257)	3.21	0.51	2.04	0.7	1.17 [1.08–1.26]	<0.001
Advise (250)	3.06 2.81	0.96	1.99	0.69	1.08 [0.98–1.18]	<0.001
Agree (234)		0.83	1.76	0.78	1.05 [0.94–1.16]	<0.001
Assist (260)	3.03	0.87	2.04	0.7	0.98 [0.88–1.09]	<0.001
Arrange (249)	2.7	0.81	1.62	0.7	1.08 [0.99–1.18]	<0.001
Overarching competencies (253)	3.22	0.88	2.77	0.97	0.44 [0.37–0.51]	<0.001
**Total^**	**18.34**	**2.66**	**12.03**	**3.2**	**6.30 [5.91–6.69]**	<0.001

*Subscale scores range 0–4; **^**Scale scores range 0–24;.

The ‘Overall competencies’ subscale, comprising a supportive attitude, had the highest average self-efficacy score with a mean (SD) of 3.22 (0.88) (Table 2). The performance score was lower, with a mean (SD) of 2.77 (0.97), but it was still the highest among the performance scores. The ‘Arrange’ subscale focuses on coordinating care and collaboration with other healthcare professionals, reflecting how nurses perceive their part in the patient's journey. This subscale showed a mean (SD) of 2.70 (0.81) for self-efficacy, indicating moderate confidence in their ability to ensure follow-up care. This was the lowest mean score compared to all the subscales of self-efficacy. Also the performance score on this subscale was the lowest score, with a mean (SD) of 1.62 (0.70) (Table 2). The ‘Assist’ subscale involves enabling patients to adapt their activities to manage living with a chronic condition. This subscale showed mean (SD) scores for self-efficacy of 3.03 (0.87) and 2.04 (0.70) for performance. The highest scores were related to questions referring to practical patient activities that directly affect nursing practice, such as encouraging patients to perform as many daily living activities as possible (see Table 3).

**Table 3:** Scores on self-efficacy and performance per subscale.

Item	Self-efficacy			Performance		
Subscale Asses	n	Mean	SD	n	Mean	SD
1. Asking the patient what he expects from living with the condition in the near future	268	3.16	0.70	267	1.71	0.98
2. Asking the patient what he knows about his condition	267	3.36	0.67	269	2.09	0.95
3. Asking the patient about how he can share his emotions about the condition with important others	266	3.18	0.68	267	1.97	1.01
4. Asking the patient about available motivation and discipline to integrate the condition into his life	267	2.96	0.77	268	1.71	1.02
5. Asking the patient how much confidence he has in his own abilities	268	3.13	0.68	264	1.88	0.96
6. Asking the patient what he can and will do in his daily healthcare	266	3.46	0.63	269	2.75	1
Subscale Advise	n	Mean	SD	n	Mean	SD
7. During each contact, asking the patient what information he needs	267	2.9	0.68	267	1.94	0.96
8. Asking the patient for permission before giving information or advice	266	3.06	0.67	265	1.92	1.04
9. Letting the patient restate the information that I gave	266	3	0.74	268	1.81	1.03
10. Giving the patient information and instruction about the condition	266	3.18	0.67	265	2.68	0.85
11. Helping the patient to formulate questions to discuss with other healthcare professionals	265	2.77	0.83	267	1.44	1
12. Involving the family when providing information and instruction	266	3.12	0.67	267	2.02	0.97
Subscale Agree	n	Mean	SD	n	Mean	SD
13. Helping the patient to identify earlier positive experiences with achieving goals	267	2.72	0.70	267	1.60	0.95
14. Allowing the patient to determine his own priorities when developing goals	266	2.68	0.74	268	1.54	0.96
15. Jointly with the patient, developing a plan of action to achieve the goals	266	2.51	0.83	268	1.27	0.95
16. Documenting the goals and agreements in the patient's record	264	2.94	0.85	265	2.06	1.25
17. Helping the patient to make decisions concerning his treatment jointly with me and/or the other healthcare professionals	266	2.77	0.73	267	1.79	1.02
18. Recognising the patient's anxiety about making a treatment decision	249	2.99	0.68	247	2.18	0.95
Subscale Assist	n	Mean	SD	n	Mean	SD
19. Discussing with the patient whom he will inform about his condition	268	2.78	0.79	265	1.23	1.06
20. Encouraging the patient to perform as many daily living activities as possible	268	3.40	0.58	269	2.96	0.86
21. Helping the patient to choose the activities that he can realistically perform	267	3.09	0.62	267	2.40	0.94
22. Discussing with the patient who (i.e., family, friends, network) can provide daily support	267	3.15	0.66	267	2.41	0.98
23. Discussing with the patient how he can make use of self-management assistive devices (i.e., diary) in his daily activities	268	2.57	0.96	269	1.25	1.09
24. Assisting the patient to monitor his own health and physical reactions	268	2.86	0.75	269	1.87	1.01
Subscale Arrange	n	Mean	SD	n	Mean	SD
25. Asking the patient about a suitable moment and a suitable approach for follow-up care	266	2.89	0.68	265	2.09	1.04
26. Consulting and making mutual plans with other healthcare professionals	268	3.13	0.65	268	2.55	0.95
27. Using assistive devices and technology (i.e., e-health) to provide remote guidance to the patient	265	1.88	1.11	265	0.47	0.92
28. Facilitating the patient to easily stay in contact between appointments	263	3.00	0.82	262	2.21	1.24
29. Initiating contact between appointments with the patient, to discuss his health and to solve possible difficulties	263	2.61	1.09	264	1.13	1.29
30. Together with the patient, examining progress of the care plan actions	260	2.58	0.95	262	1.18	1.05
Overarching competencies	n	Mean	SD	n	Mean	SD
31. Acknowledging the patient's experiential knowledge as valuable information concerning my own care delivery	264	3.14	0.66	262	2.56	1.01
32. Considering the (cultural) background of the patient	265	3.22	0.66	266	3.00	0.85
33. Together with the patient, determining how much of the care coordination I take over for him	264	3.16	0,64	265	2.68	1.02
34. Using the patient's choice as the basis for care, even if it is not ideal from a medical perspective	263	2.88	0.76	266	2.24	0.97
35. Showing understanding when the patient does not succeed in achieving the established goals	265	3.29	0.61	267	2.84	0.91
36. Reflecting on my own management (of care)	265	3.26	0.65	266	2.98	0.91

### 3.3 Interviews

The qualitative analyses revealed three major themes on barriers and facilitators for self-management support: ‘Routines and priorities’ ‘Goal setting and aftercare’, and ‘The role of nurses in hospital care’. In the following sections, we discuss the interviews based on these themes.

#### 3.3.1 Routines and priorities

The interviews revealed that an important factor influencing nurses' support for patients' self-management is their tendency to work according to routines and protocols. They work with a mental checklist making sure they do check all the boxes. Nurses commented that they hardly consider supporting patients’ self-management during their shifts. As a result, they focus mainly on functional tasks such as wound care and ambulation, leaving scarce opportunity for individual approaches.

*"Some patients prefer to bathe in the evening. I believe that individuals should have the autonomy to make their own choices in this matter. Our current approach to care adheres to a rigid structure. This has become ingrained, and I feel it's important for us to be more mindful and analytical of this from time to time. Do tasks truly need to be completed in a specific manner, or is there room for a different approach?"* (P33)

When patients can take care of themselves or are medically out of treatment, they may receive less attention from nurses because their needs are less visible. Assessing patients’ experiences about living with the condition was often regarded as a surplus. Typically, only the most critical medical information was collected from the patients. Nevertheless, some participants argued that assessment of patients' experiences should be prioritised and suggested it should be a ‘check box activity’, like taking temperatures or assessing pain. They argued that eliciting information about patients' living conditions would help them achieve early discharge goals because the patients would learn how to cope with the condition by themselves. Nevertheless, nurses seemed to run from task to task, not taking time for meaningful interaction with patients. However, some participants resisted the culture that prioritises production and checklists over patient care.

*“I also think you must make some time for it. The patient is and remains a human being, so you must keep seeing him as a human rather than an object. I think that would be the end, and you would just take all the control out of your hands. You think, "Well, I don't have time, so I just do everything: washing and dressing, and I throw them back in bed, and I don't take them out again”. If you start doing that, the nursing profession drops out of sight; you lose the nursing profession out of the picture. Then you're running a car wash more than helping someone get better”.* Oncology ward nurse (P9)

When interviewed, participants were asked about their perspectives on patient care and the implementation of their ideals in daily practice. Many admitted that they did not always adhere to their ideals, despite recognising the importance of psychosocial support in patient care. They attributed this to factors such as lack of time, a focus on technical aspects, or difficulty initiating in-depth conversations with patients. Nevertheless, they acknowledged that better communication with patients leads to more purposeful work and greater job satisfaction.

Some participants admitted that they would not engage in-depth conversations with patients even if they had more time available. They mentioned it was simply not their inclination or that they valued moments of quiet, albeit with some guilt. Others, however, would take the time to educate patients on self-care for the future at home or to spend more time with them.

*“I think it's mostly up to yourself. If you believe it's important and someone asks why you're sitting next to the patient's bedside talking while you're explaining something to the patient, then you don't feel encumbered by that, and you can defend yourself about it just fine. But you won't do that if you don't understand why it might be important*.” Gastrointestinal ward nurse (P21)

#### 3.3.2 Goal setting and aftercare

The interviewees admitted having mixed feelings about goal setting. While they disliked the administrative burden and time commitment, many acknowledged its benefits. Benefits included facilitating earlier discharge and, in some cases, eliminating the need for home care by better-preparing patients and their families to provide care.

“*I believe the patient also goes home more satisfied. Suppose someone comes in with heart failure, and we have dehydrated him. Yes, the patient likes that; they no longer have oedema or tightness of the chest. But nothing else has changed during that admission. I think that can sometimes feel hopeless for a patient.”* Cardiopulmonary ward nurse (P35)

Participants emphasised the importance of monitoring progress when setting goals with patients, noting that documentation in patient records is crucial for continuity. However, building a trusting relationship and having in-depth conversations with patients was challenging due to irregular shifts. Consequently, since patients were only briefly admitted to the ward, nurses did not often invest time in goal setting.

The interviewees primarily mentioned goals related to functional activities, such as mobilising and engaging in daily living and care activities. Very few mentioned goals related to the psychosocial aspects of coping with a condition. While many nurses were not accustomed to setting goals themselves, they were familiar with working towards goals offset by other healthcare professionals, such as physiotherapists.

Providing information about recognising symptoms and instructions about the medical regimen is part of supporting patients’ self-management. Participants emphasised that providing education is more effective when tailored to the individual patient. However, nurses often provide information through generic leaflets without personalisation or confirmation that it meets patients’ specific needs. Participants acknowledged that hospital admissions would be more valuable if nurses used this time to help patients learn how to monitor their health at home.

“*Of course, it is effortless to give a leaflet with the instructions 'please read it and if necessary, you can call this number'. Then you're done within ten seconds. […]. However, the downside is that when a patient has a language barrier or can't read, there's no point. Then, the patient is completely unaware that he has to weigh himself at home, for example. Of course, if you sit down with the family or whoever is in the network and say: 'OK, who can keep an eye on this gentleman weighing himself or who can help him with that?’ The network also knows that man or woman must stick to those precepts.”* Cardiology ward nurse (P5)

#### 3.3.3 The role of nurses in hospital care

Many interview participants emphasised that nurses should play a central role in the care process because they know the patients best and even have insight into the home situation, unlike physicians. In addition, nurses are approachable contacts for other disciplines. Despite this, when discussing their collaboration with other disciplines, participants used passive language, such as: “*We're not asked to be present”* or “*Physicians do not ask for our opinion”*. This indicates that nurses perceive their roles and tasks as largely dictated by others and often act mainly as facilitators for other professions. While in some wards, nurses and physicians excellently collaborate, in other wards, nurses must make more effort to discuss patient issues with physicians. Participants felt that nurses need to advocate more for their profession and establish their own rules instead of merely implementing those of other disciplines.

*“I think that sometimes tasks are just thrown at our heads. Like: do this too. […] the nurse is often more of a worker bee than a spider in the web.”* Oncology ward nurse (P8)

While the central role of nurses can be an important facilitator for self-management support, the role that patients expect from nurses could hamper this support. The participants experienced that patients and nurses often hold different expectations regarding their roles, which are rarely discussed during the admission interview. Patients typically expect that hospital admission will lead to improvement or treatment of their illness. According to the participants, they often rely on nurses for the simplest tasks, even when they are able to do these themselves. This reliance has led some nurses to view these patients as lazy or inactive, feeling taken advantage of.

*“Older patients or people who have been in hospital more often are more likely to be dependent. They think: ‘I'm in hospital, so those nurses will help you’. Well, if you haven't had surgery on your hands, then you can do it yourself, washing your face.”* (P29)

Other participants believed that keeping patients as active as possible benefits the patients and saves nurses time. Patients are expected to be actively involved in their care, but the nurses’ time constraints often limit their ability to do so. When nurses are busy, they tend to take over tasks from patients to save time, which can give the impression that nurses are determining the extent of patient involvement in their daily care.

## 4 Discussion

This study provided insight into the challenges nurses face in supporting patients’ self-management within a hospital environment. Despite nurses expressing confidence in their ability to offer such support, the results of our questionnaire study revealed a statistically significant gap between this assertion and the actual support provided in practice, as reported by the nurses themselves. This finding echoes similar results from previous studies utilising the SEPSS-36 (Duprez et al., 2021; van Hooft et al., 2016). Moreover, a study on person-centred care has shown a discrepancy between nurses’ perceptions of their provision of person-centred care and their observed actual behaviour when observed (Bolster and Manias, 2010). Although self-efficacy has proven to be a significant factor in performing self-management activities (van Hooft et al., 2016), explanations for nurses’ behaviour can be found in other factors. Michie et al. (2011) distinguish six components influencing behaviour: *Physical* and *Psychological Capability, Physical* and *Social Opportunity*, and *Reflexive* and *Automatic processes* (*Motivation*). These components interact and are all necessary for specific behaviour. We saw the components *Physical* and *Social Opportunity*, and *Motivation* and *Capability* reflected in nurses’ interview responses. Factors impeding self-management support within the hospital setting were nurses’ routines with an emphasis on medical management, the expectations of both nurses and patients towards their roles in the care process, and a short-term mindset. We also found the opportunity for nurses to have a central position in patient care, which can enhance self-management, provided nurses are sufficiently empowered to take this role.

The nurses who participated in this study primarily focused on the medical management part of self-management, which aligns with previous studies (van Belle et al., 2020; Feo & Kitson, 2016). Nurses tend to work with a mental checklist, prioritising medical tasks and maintaining patients’ functional status. Spending supportive time with patients is often considered an additional task, which is one of the first to be neglected in the presence of time constraints (Ausserhofer et al., 2014). While emotional labour as defined by Jackson et al. (2021), is an essential part of the nursing profession, managers still do not ordinarily acknowledge this for fear of interfering with efficiency (Traynor, 2019). Still, neglecting supportive time with patients could eventually lead to burnout and leaving the nursing profession (Stemmer et al., 2022), seeing that nurses generally consider personal contact with patients the most rewarding aspect of their job (Morgan and Lynn, 2009). In our study, nurses provided examples of engaging in-depth conversations with patients during daily care, consistent with van Belle et al. (2020) findings that such interactions can occur even in busy hospital settings. However, high patient turnover and lack of continuity due to irregular shifts were identified as barriers to having such conversations with patients regularly.

We noted that many nurses prioritise the goals offset by other healthcare professionals, such as physiotherapists, over setting goals with patients about daily activities at home. Suhonen et al. (2018) also found that prioritisation of care is not always based on nursing care requirements but rather on medical requirements.

Another factor influencing self-management support is the mutual expectations of patients and nurses regarding each other's roles. According to the nurses in this study, patients often expect to be taken care of during admission. In contrast, the nurses encourage patients to do as much as possible as they would at home, even though nurses sometimes take over. These differing expectations are not discussed during patient admission, although such discussions could clarify the roles and prevent nurses from doing too little or too much (Ingstad et al., 2023).

This study found that a perceived lack of time is not the only constraint on supporting self-management. Many participants acknowledged that they would not provide more support, even if they had time for it. Their mindset is focused on immediate nursing tasks, making it more challenging to address emotional or role management issues (Lorig and Holman, 2003). Questions about the patient's home situation or motivations often lie beyond the scope of the short hospital admission. Addressing these issues demands actions in all phases of the Five A's Model of self-management support (Glasgow et al., 2003). Our study identified also room for improvement in the ‘Arrange’ phase, which involves follow-up care. Consistent with a recent study on self-management support in hospitalised patients (Otter et al., 2022), we found that nurses often fail to focus on how the patients will manage after discharge. However, recognising the importance of understanding what patients need to maintain their health at home is crucial (Fishbein et al., 2019).

Another opportunity for self-management support lies in the benefits of mutual goal setting, although the nurses in our study revealed ambiguous feelings about it. In order to profit from the benefits, a focus on the bigger picture rather than just short-term priorities is a necessity. While the outcomes of coordinating activities and reporting goals may not be immediately visible, they are essential components of a nurse's work (Jackson et al., 2021). This is often referred to as the "invisible work" of nurses (Allen, 2014). By setting goals with patients and actively working towards them, nurses can make a meaningful impact with long-term benefits. Ultimately, this can lead to decreased workloads for nurses in both ward and home care settings (Kelly, 2011; Klooster et al., 2022; Verkerk et al., 2023).

### 4.1 Strengths and limitations

Our study achieved a high response rate of 62 %, encompassing many wards of the participating hospital. However, it is important to note that the interview sample was a convenience sample, potentially introducing selection bias. Nurses confident in supporting self-management may have been more likely to participate. Moreover, nurse students collected the data for the interviews as part of their bachelor's thesis and recruited the participants themselves. As a result, they might have interviewed nurses who were ‘approachable’ and willing to participate.

It is also worth noting that the questionnaire's outcomes were based on self-reported data. Therefore, we cannot be certain that these findings reflect the actual self-management support provided by nurses. However, the interviews also revealed a discrepancy between the nurses' ability to support self-management and the actual implementation of such support in their daily practice.

This study was executed in one hospital in one country, which may impact its generalisability to other institutions and countries. However, the findings align with other studies in hospital settings in the Netherlands (van Belle et al., 2020; Otter et al., 2021).

## 5 Implications for clinical practice and future research

The study shows that many nurses are willing to support patients’ self-management but that, due to time constraints, daily routines dominate their work. Since self-management support is to the benefit of healthcare organisations as well, managers could put more value in psychosocial care and reflect on the organisation of work.

Future research could study the effect of mutual goal-setting by nurses and patients on readmission and the level of care required. As this study centres on the perspectives of nurses, there is uncertainty regarding the experiences of patients. It would be valuable to evaluate patients' perceptions of the self-management support provided by nurses in a hospital environment.

## 6 Conclusions

In this study, many nurses have emphasised the importance of engaging in-depth conversations with patients and providing psychosocial care. However, they acknowledge that their daily practice primarily focuses on fulfilling their immediate duties and 'getting the job done'. This focus often leads to failure to recognise the benefits of supporting patient self-management, particularly in units with high patient turnover. This limited perspective on their responsibilities may impede patient care, whereas adopting a broader view on the patients' journey could be highly beneficial. Conducting a thorough assessment of the patient's situation and motivation at the outset could potentially decrease readmissions and reduce workloads in other healthcare settings.

## Funding sources

Funded by the Netherlands Organisation for Health Research and Development (ZonMw): 80-86300-98-124.

## CRediT authorship contribution statement

**Susanne van Hooft:** Writing – original draft, Supervision, Project administration, Formal analysis, Data curation, Methodology, Conceptualization. **Elke Berger:** Writing – review & editing, Supervision, Project administration, Investigation, Formal analysis, Data curation. **Cailey van Torenburg:** Writing – review & editing, Investigation, Formal analysis, Data curation. **AnneLoes van Staa:** Writing – review & editing, Supervision, Methodology, Conceptualization.

## Declaration of competing interest

The authors declare that they have no known competing financial interests or personal relationships that could have appeared to influence the work reported in this paper.

## Appendix Supplementary materials

Image, application 1

Image, application 2

## References

[bib0001] Allen D. (2014).

[bib0002] Ausserhofer D., Zander B., Busse R., Schubert M., De Geest S., Rafferty A.M., Ball J., Scott A., Kinnunen J., Heinen M. (2014). Prevalence, patterns and predictors of nursing care left undone in European hospitals: results from the multicountry cross-sectional RN4CAST study. BMJ Qual. Saf..

[bib0003] Bandura A. (2006).

[bib0004] Barlow J., Wright C., Sheasby J., Turner A., Hainsworth J. (2002). Self-management approaches for people with chronic conditions: a review. Patient. Educ. Couns..

[bib0005] Been-Dahmen J.M., Dwarswaard J., Hazes J.M., van Staa A., Ista E. (2015). Nurses' views on patient self-management: a qualitative study. J. Adv. Nurs..

[bib0007] Bolster D., Manias E. (2010). Person-centred interactions between nurses and patients during medication activities in an acute hospital setting: qualitative observation and interview study. Int. J. Nurs. Stud..

[bib0008] Brand C.A., Barker A.L., Morello R.T., Vitale M.R., Evans S.M., Scott I.A., Stoelwinder J.U., Cameron P.A. (2012). A review of hospital characteristics associated with improved performance. Int. J. Qual. Health Care.

[bib0009] Creswell J.W., Clark V.P. (2011).

[bib38] Creswell J.W., Creswell J.D. (2017).

[bib0010] Duprez V., Van Hooft S.M., Dwarswaard J., van Staa A., Van Hecke A., Strating M.M. (2016). The development and psychometric validation of the self-efficacy and performance in self-management support (SEPSS) Instrument. J. Adv. Nurs..

[bib0011] Duprez V., Vansteenkiste M., Beeckman D., Verhaeghe S., Van Hecke A. (2021). Capturing motivating versus demotivating self-management support: development and validation of a vignette-based tool grounded in Self-determination Theory. Int. J. Nurs. Stud..

[bib0012] Elissen A., Nolte E., Knai C., Brunn M., Chevreul K., Conklin A., Durand-Zaleski I., Erler A., Flamm M., Frolich A., Fullerton B., Jacobsen R., Saz-Parkinson Z., Sarria-Santamera A., Sonnichsen A., Vrijhoef H. (2013). Is Europe putting theory into practice? A qualitative study of the level of self-management support in chronic care management approaches. BMC. Health Serv. Res..

[bib39] Feo R., Kitson A. (2016). Promoting patient-centred fundamental care in acute healthcare systems. Int. J. Nurs. Stud..

[bib0013] Fishbein D., Nambiar S., McKenzie K., Mayorga M., Miller K., Tran K., Schubel L., Agor J., Kim T., Capan M. (2019). Objective measures of workload in healthcare: a narrative review. Int. J. Health Care Qual. Assur..

[bib40] Glasgow R.E., Davis C.L., Funnell M.M., Beck A. (2003). Implementing practical interventions to support chronic illness self-management. Jt Comm. J. Qual. Saf..

[bib0014] Glasgow R.E., Emont S., Miller D.C. (2006). Assessing delivery of the five 'As' for patient-centered counseling. Health Promot. Int..

[bib0015] Ingstad K., Pedersen M.K., Uhrenfeldt L., Pedersen P.U. (2023). Patients’ expectations of and experiences with psychosocial care needs in perioperative nursing: a descriptive study. BMC. Nurs..

[bib0016] Jackson J. (2021). What is nursing work? A meta-narrative review and integrated framework. Int. J. Nurs. Stud..

[bib0017] Kelly M.D. (2011). Self-management of chronic disease and hospital readmission: a care transition strategy. J. Nurs. Healthc. Chronic. Illn..

[bib0018] Kendall E., Ehrlich C., Sunderland N., Muenchberger H., Rushton C. (2011). Self-managing versus self-management: reinvigorating the socio-political dimensions of self-management. Chronic. Illn..

[bib0020] Klooster E., Koenders N., Vermeulen-Holsen J., Vos L., van der Wees P.J., Hoogeboom T.J. (2022). Healthcare professionals feel empowered by implementing a hospital-based multifaceted intervention: a qualitative study using inductive thematic analysis. BMC. Health Serv. Res..

[bib0021] Kosmala-Anderson J.P., Wallace L.M., Turner A. (2010). Confidence matters: a self-determination theory study of factors determining engagement in self-management support practices of UK clinicians. Psychol. Health Med..

[bib0022] Lorig K.R., Holman H. (2003). Self-management education: history, definition, outcomes, and mechanisms. Ann. Behav. Med..

[bib0023] Michie S., Van Stralen M.M., West R. (2011). The behaviour change wheel: a new method for characterising and designing behaviour change interventions. Implement. Sci..

[bib0024] Morgan J.C., Lynn M.R. (2009). Satisfaction in nursing in the context of shortage. J. Nurs. Manag..

[bib0025] Noordman J., Meurs M., Poortvliet R., Rusman T., Orrego-Villagran C., Ballester M., Ninov L., de Guzmán E.N., Alonso-Coello P., Groene O., Suñol R., Heijmans M., Wagner C. (2023). Contextual factors for the successful implementation of self-management interventions for chronic diseases: a qualitative review of reviews. Chronic. Illn..

[bib0026] Otter C.E., Keers J.C., Reker C., Smit J., Schoonhoven L., de Man-van Ginkel J.M. (2022). How nurses support self-management of hospitalized patients through verbal communication: a qualitative study. BMC. Nurs..

[bib0027] Otter C.E., Smit J., Hagedoorn E.I., Keers J.C., de Man-van Ginkel J.M., Schoonhoven L. (2021). Nurses’ perceptions of self-management and self-management support of older patients during hospitalization. Geriatr. Nurs. (Minneap).

[bib0028] Rosnawati M.R., Mohd Fauzi M.F., Mat Saruan N.A., Mohd Yusoff H., Harith A.A. (2021). Why so stressed? A comparative study on stressors and stress between hospital and non-hospital nurses. BMC. Nurs..

[bib0029] Stavropoulou A., Rovithis M., Kelesi M., Vasilopoulos G., Sigala E., Papageorgiou D., Moudatsou M., Koukouli S. (2022). What quality of care means? Exploring clinical nurses’ perceptions on the concept of quality care: a qualitative study. Clin. Pract..

[bib0030] Stemmer R., Bassi E., Ezra S., Harvey C., Jojo N., Meyer G., Özsaban A., Paterson C., Shifaza F., Turner M.B., Bail K. (2022). A systematic review: unfinished nursing care and the impact on the nurse outcomes of job satisfaction, burnout, intention-to-leave and turnover. J. Adv. Nurs..

[bib0031] Suhonen R., Stolt M., Habermann M., Hjaltadottir I., Vryonides S., Tonnessen S., Halvorsen K., Harvey C., Toffoli L., Scott P.A. (2018). Ethical elements in priority setting in nursing care: a scoping review. Int. J. Nurs. Stud..

[bib0032] Tharani A., Van Hecke A., Ali T.S., Duprez V. (2021). Factors influencing nurses’ provision of self-management support for patients with chronic illnesses: a systematic mixed studies review. Int. J. Nurs. Stud..

[bib0033] Traynor M. (2019). Autonomy and caring: towards a Marxist understanding of nursing work. Nurs. Philosophy.

[bib0006] van Belle E., Giesen J., Conroy T., Mierlo M., Vermeulen H., Huisman-de Waal G., Heinen M. (2020). Exploring person-centred fundamental nursing care in hospital wards: a multi-site ethnography. J. Clin. Nurs..

[bib0034] van Hooft S.M., Becqué Y.N., Dwarswaard J., van Staa A., Bal R. (2018). Teaching self-management support in Dutch bachelor of nursing education: a mixed methods study of the curriculum. Nurse Educ. Today.

[bib0035] Van Hooft S.M., Dwarswaard J., Bal R., Strating M.M., van Staa A. (2016). What factors influence nurses' behavior in supporting patient self-management? An explorative questionnaire study. Int. J. Nurs. Stud..

[bib0036] Verkerk E.W., Boekkooi J.A.H., Pels E.G.M., Kool R.B. (2023). Exploring patients’ perceptions of low-value care: an interview study. Patient. Educ. Couns..

[bib0037] Whitlock E.P., Orleans C.T., Pender N., Allan J. (2002). Evaluating primary care behavioral counseling interventions: an evidence-based approach. Am. J. Prev. Med..

