# Supplementary material for: Daily routines, short-term priorities, and nurses’ role hamper self-management support in a hospital setting: A mixed methods study
Source: Int J Nurs Stud Adv. 2024 Dec 5;8:100279. doi: 10.1016/j.ijnsa.2024.100279 (PMC11667052; doi:10.1016/j.ijnsa.2024.100279)
Supplement: Supplementary file 1 [file mmc1.docx]

The respondents were assured raw data would remain confidential and would not be shared.

Data not available / The data that has been used is confidential
