# Supplementary material for: Daily routines, short-term priorities, and nurses’ role hamper self-management support in a hospital setting: A mixed methods study
Source: Int J Nurs Stud Adv. 2024 Dec 5;8:100279. doi: 10.1016/j.ijnsa.2024.100279 (PMC11667052; doi:10.1016/j.ijnsa.2024.100279)
Supplement: Supplementary file 2 [file mmc2.pdf]

Francıscus
Gasthuis & Vlietland

Franciscus Gasthuis & Vlietland
Mw. E. Berger
Wetenschapsbureau
Locatie Gasthuis

**Afdeling**	Raad van Bestuur
**Telefoon**	010-461 6005
**Onze referentie**	T 110
**Rotterdam**	17 maart 2021
**Betreft onderzoek**	De rol van de verpleegkundige bij zelfmanagement ondersteuning, Zelfmanagement ondersteuning

Onderwerp: Toestemming Raad van Bestuur niet-WMO plichtige studie met het studienummer 2021-044-z

Geachte mevrouw Berger,

Hierbij laat ik u weten dat de Raad van Bestuur van het Francíscus Gasthuis & Vlietland kennis heeft genomen van voorgenoemd onderzoeksprotocol en toestemming verleent voor de uitvoering van bovengenoemde onderzoek in haar instelling. U dient hierbij te werken volgens de Nederlandse gedragscode wetenschappelijke integriteit.

De Raad van Bestuur baseert deze verklaring op de volgende overwegingen:

- De deskundigheid en bekwaamheid van lokale onderzoekers en ondersteunend personeel van het onderzoek;
- Het beroepsmatig inlichten van alle personen, die een bijdrage moeten leveren aan het onderzoek over het onderzoeksprotocol;
- De geschiktheid van de faciliteiten en de instelling voor een gedegen uitvoering van het onderzoek;
- De niet gelijktijdige uitvoering van andere onderzoeken in dezelfde instelling, waardoor het welslagen van het onderzoek en van andere onderzoeken bemoeilijkt kan worden;
- Het positieve advies en evt. bijbehorende voorwaarden van de Advies Commissie Wetenschap en de aangeleverde documenten (zie bijlage 1).

Het gebruik van de STZ-Standard Operating Procedures (SOPs) is verplicht bij de uitvoering van wetenschappelijk onderzoek in ons ziekenhuis. Deze kunt u vinden via intranet:
Franciscus ABC > W > Wetenschapsbureau > Procedures en formats.
Gebruik van SOPs dient u vast te leggen in de trial master file middels VL1 FRM_02b Taakverdeling Franciscus geïnitieerd single-center onderzoek.

Locatie Gasthuis
Kleiweg 500, 3045 PM Rotterdam
tel. 010 - 461 6161

Locatie Vlietland
Vlietlandplein 2, 3118 JH Schiedam
tel. 010 - 893 9393

www.franciscus.nl
K.v.K. nummer 56474113

franciscus
Gasthuis & Vlietland

Tevens attenderen wij u op het volgende:

- Voor deze studie worden geen extra studieverrichtingen in HiX geregistreerd.
- Castor SMS dient up-to-date te blijven en fungeert als digitale deel van het studiedossier (TMF). Meldt in Castor SMS de volgende zaken:
    - amendementen (tabblad amendementen)
    - jaarlijkse voortgang en/of eindrapportage (tabblad studie voortgang)
- Bij niet-WMO plichtig onderzoek mogen onderzoeken geregistreerd worden in een erkend trial register (zie ook STZ SOP VC7 'Aanmelding trialregister'), maar is niet verplicht.
- In de bijlage is de niet-WMO verklaring van de Adviserende Commissie Wetenschap bijgevoegd (bijlage 2).

Voor vragen rondom gebruik van SOPs of andere vragen t.a.v. wetenschappelijk onderzoek kunt u terecht bij het Wetenschapsbureau (wetenschapsbureau@franciscus.nl / 010-461 7155).

Wij wensen u veel succes met de uitvoering van het onderzoek.

Met vriendelijke groeten, namens de Raad van Bestuur

dr. M.J.A. Tasche, voorzitter Raad van Bestuur

CC:
wetenschapsbureau@franciscus.nl

franciscus
Gasthuis & Vlietland

Bijlage 1. Advies lokale uitvoerbaarheid *Advies Commissie Wetenschap* (ACW) Franciscus

Intern studienummer: 2021-044-z

Geacht lid Raad van Bestuur,

Mw. E. Berger, coördinator zorgonderzoek, heeft via Castor SMS de volgende studie ingediend voor toestemming voor uitvoer in onze instelling.

Titel studie:	De rol van de verpleegkundige bij zelfmanagement ondersteuning
Korte titel:	Zelfmanagement ondersteuning
METC:	ACW FG&V
METC nr van de studie:	ACW getoetst
WMO plichtig:	Nee
Initiator:	Franciscus geïnitieerd
Opdrachtgever/Verrichter:	Raad van Bestuur

Korte samenvatting studie (doel):
Het ondersteunen van zelfmanagement wordt in het beroepsprofiel beschreven als de kern van verplegen. Ook veel patiëntenorganisaties benadrukken het belang van goed zelfmanagement. Toch blijkt het voor veel verpleegkundigen in de praktijk niet altijd even eenvoudig om hier handen en voeten aan te geven. Het is niet duidelijk in hoeverre verpleegkundigen op dit moment zelfmanagement ondersteuning bieden in de dagelijkse zorg. Wat denken verpleegkundigen te kunnen en doen in de praktijk bij zelfmanagement ondersteuning. Wat zijn situaties waarin het juist wel goed lukt of niet en vooral ook welke ruimte durven verpleegkundigen in te nemen (prioriteiten stellen, ingaan tegen anderen, leiderschap dus).

De commissie ACW heeft de lokale uitvoerbaarheidsprocedure doorlopen. De studiedocumenten van lokale hoofdonderzoeker zijn gecontroleerd en geborgd in Castor SMS.
Financiële afspraken zijn vastgelegd in het financiële overzicht dd. 17 maart 2021

De commissie ACW heeft geen bezwaar tegen uitvoering van dit onderzoek. Derhalve adviseren wij positief over de uitvoering van deze studie in ons ziekenhuis onder de volgende overeengekomen voorwaarden:

Voorwaarden:

- De toestemming wordt verleend op basis van de documentatie die geüpload is in Castor SMS en een goedkeuringsstatus heeft ontvangen (status 'akkoord door Wetenschapsbureau of 'goedgekeurd door METC'). Indien nieuwe documentatie wordt geüpload, houdt dit niet in dat deze automatisch goedgekeurd is. Door amendementen te melden in Castor SMS kunnen de nieuwe documenten tussentijds worden goedgekeurd. Indien relevant wordt de lokale haalbaarheid hierbij mede her-beoordeeld en bijgesteld.

Met vriendelijke groet,

De Adviescommissie Wetenschap

dr. B.M. Boxma-de Klerk, mw. A.L. Hanschke, mw. J.S. Mills, mw. F.J. van den Oever en dr. G.J. Braunstahl.

Franciscus Gasthuis & Vlietland

Bijlage 2. Niet WMO verklaring *Advies Commissie Wetenschap* (ACW) Franciscus

Intern studienummer: 2021-044-z

Titel studie: De rol van de verpleegkundige bij zelfmanagement ondersteuning
Korte titel: Zelfmanagement ondersteuning

Betreft: Protocol versie: <1, 9 maart 2021>

Bovengenoemd onderzoek is door de lokale Advies Commissie Wetenschap (ACW) beoordeeld als niet-WMO plichtig:

Aanvraag betreft een afstudeeropdracht voor HBO-V studenten. Doel van het onderzoek is het achterhalen op welke wijze verpleegkundigen op dit moment zelfmanagement ondersteuning bieden in de dagelijkse zorg. Wat denken verpleegkundigen te kunnen en doen in de praktijk bij zelfmanagement ondersteuning. Onder verpleegkundigen van de betreffende afdeling wordt een korte vragenlijst verspreid en interviews afgenomen. Deze zijn kort en bevatten geen vragen die de persoonlijke levenssfeer kunnen schaden. Dit project zal op verschillende afdelingen in het FG&V worden uitgevoerd. Deze studie is niet-WMO plichtig.

To whom it may concern:

The study mentioned above was reviewed by the Institutional Review Board (ACW) of the Franciscus Gasthuis & Vlietland. As a result of this review, the ACW informs you that the rules laid down in the Medical Research Involving Human Subjects Act (also known by its Dutch abbreviation WMO), do not apply to this research proposal. The study was approved by the Board of Directors of the Franciscus Gasthuis & Vlietland. Data handling is compliant with the GDPR.

17 MRT 2021
